# 

**Published:** 1882-12

**Authors:** 


### CONTENTS:

PAGE.

**ORIGINAL COMMUNICATIONS:**

I. Microscopical Investigation into the Nature of the So-called Bacillus Tuberculosis. By H. D. Schmidt, M.D. .... 561

**SOCIETY REPORTS:**

II. American Dermatological Association. Proceedings at the Sixth Annual Meeting .... 585

**DOMESTIC CORRESPONDENCE:**

III. New York Letter .... 611

Reviews and Book Notices .... 612

Books and Pamphlets Received .... 617

PAGE.

**EDITORIAL:**

English Criticisms of Americans .... 620

Dr. Schmidt's Paper on Bacillus Tuberculosis .... 625

**SELECTIONS:**

Deflection of the Septum Narium. By E. Fletcher Ingals, M.D .... 627

State Board of Health of West Virginia .... 635

Glycosuria. By Oscar C. DeWolf, M D .... 642

A Prepared Bill to Regulate the Practice of Medicine in Michigan. By Henry B. Baker, M.D .... 653

The Vomiting of Pregnancy. By Wm. B. Atkinson, M.D .... 661

ITEMS .... 611, 619, 626, 667, 668

ANNOUNCEMENTS FOR THE MONTH .... 672

NOTICE TO CORRESPONDENTS .... *advts.*

